# Diagnostic and Therapeutic Challenges in Asthma and Chronic Obstructive Pulmonary Disease Overlap: A Four-Patient Case Series

**DOI:** 10.7759/cureus.112821

**Published:** 2026-07-16

**Authors:** Benedict Amalraj, Mariana Marrero Castillo, Neerja Gulati

**Affiliations:** 1 Internal Medicine, Louisiana State University Health Sciences Center, Shreveport, USA; 2 Pulmonary Medicine, Louisiana State University Shreveport, Shreveport, USA

**Keywords:** aco: asthma-copd overlap, asthma, biologics, clinical features, copd

## Abstract

Asthma-chronic obstructive pulmonary disease (COPD) overlap is a heterogeneous clinical descriptor applied to patients with persistent airflow limitation who exhibit features of both asthma and chronic obstructive pulmonary disease. Diagnostic uncertainty is common, particularly when smoking-related emphysema coexists with asthma-associated traits such as bronchodilator reversibility, allergic rhinitis or atopy, eosinophilia, and elevated immunoglobulin E.

We report four adults with chronic obstructive physiology and overlapping clinical, radiographic, physiologic, and inflammatory features. Two patients had persistent symptoms despite guideline-directed inhaled therapy and were treated with add-on biologic therapy targeting type 2 inflammation using benralizumab or dupilumab. The dupilumab-treated case demonstrated symptomatic and spirometric improvement over three months, whereas the benralizumab-treated case illustrates phenotype-guided biologic selection in eosinophilic asthma with coexisting COPD features and persistent severe obstruction on verified follow-up spirometry. The remaining cases illustrate practical diagnostic challenges, including the interpretation of serial spirometry, the recognition of bronchodilator responsiveness, and the reconciliation of radiographic emphysema with asthma-like variability and allergic sensitization. This case series highlights the clinical heterogeneity of asthma-chronic obstructive pulmonary disease overlap and supports a phenotype-informed approach incorporating spirometry, imaging, allergic evaluation, and accessible type 2 inflammatory biomarkers. Further prospective studies are needed to define which overlap phenotypes are most likely to benefit from biologic therapy.

## Introduction

Asthma-chronic obstructive pulmonary disease (COPD) overlap (ACO) has been described using varying case definitions, contributing to heterogeneity across cohorts and complicating translation into routine clinical practice [1]. The Global Initiative for Asthma (GINA) provides a framework for asthma diagnosis and management, whereas the Global Initiative for Chronic Obstructive Lung Disease (GOLD) provides an evidence-based strategy for COPD diagnosis and treatment [2,3]. Systematic reviews suggest that patients with overlap features frequently have a higher symptom burden and may experience more frequent or more severe exacerbations than patients with asthma or COPD alone [4]. In addition, real-world observational data indicate that treatment-response patterns to biologics in selected patients with ACO may resemble those observed in asthma, supporting careful consideration of biologic therapy in appropriately phenotyped patients [5]. Prevalence estimates vary substantially by definition. In a systematic review and meta-analysis, the reported ACO prevalence ranged from 0.3% to 5.0% in the general population, 3.2% to 51.4% among patients with asthma, and 12.6% to 55.7% among patients with COPD [6].

In 2017, an American Thoracic Society/National Heart, Lung, and Blood Institute workshop emphasized that ACO should not be regarded as a single distinct disease entity, but rather as a construct encompassing multiple phenotypes and mechanisms [7]. Earlier joint GINA/GOLD documents proposed a syndromic, stepwise approach to identify chronic airway disease, confirm persistent airflow limitation by spirometry, and assess features favoring asthma, COPD, or overlap to guide treatment decisions [8]. Expert consensus discussions have likewise highlighted persistent definitional challenges and the need for clinically usable criteria that still capture the heterogeneity of overlap presentations [9]. Consistent with this framework, diagnostic labeling should not replace individualized phenotyping, particularly when smoking-related emphysema, persistent airflow limitation, bronchodilator responsiveness, allergic sensitization, eosinophilia, or elevated immunoglobulin E coexist. We present four cases illustrating diagnostic and management challenges in ACO, with particular attention to type 2 inflammatory features and the potential role of biologic therapy in carefully selected patients.

## Case presentation

Case 1

**Figure 1:**
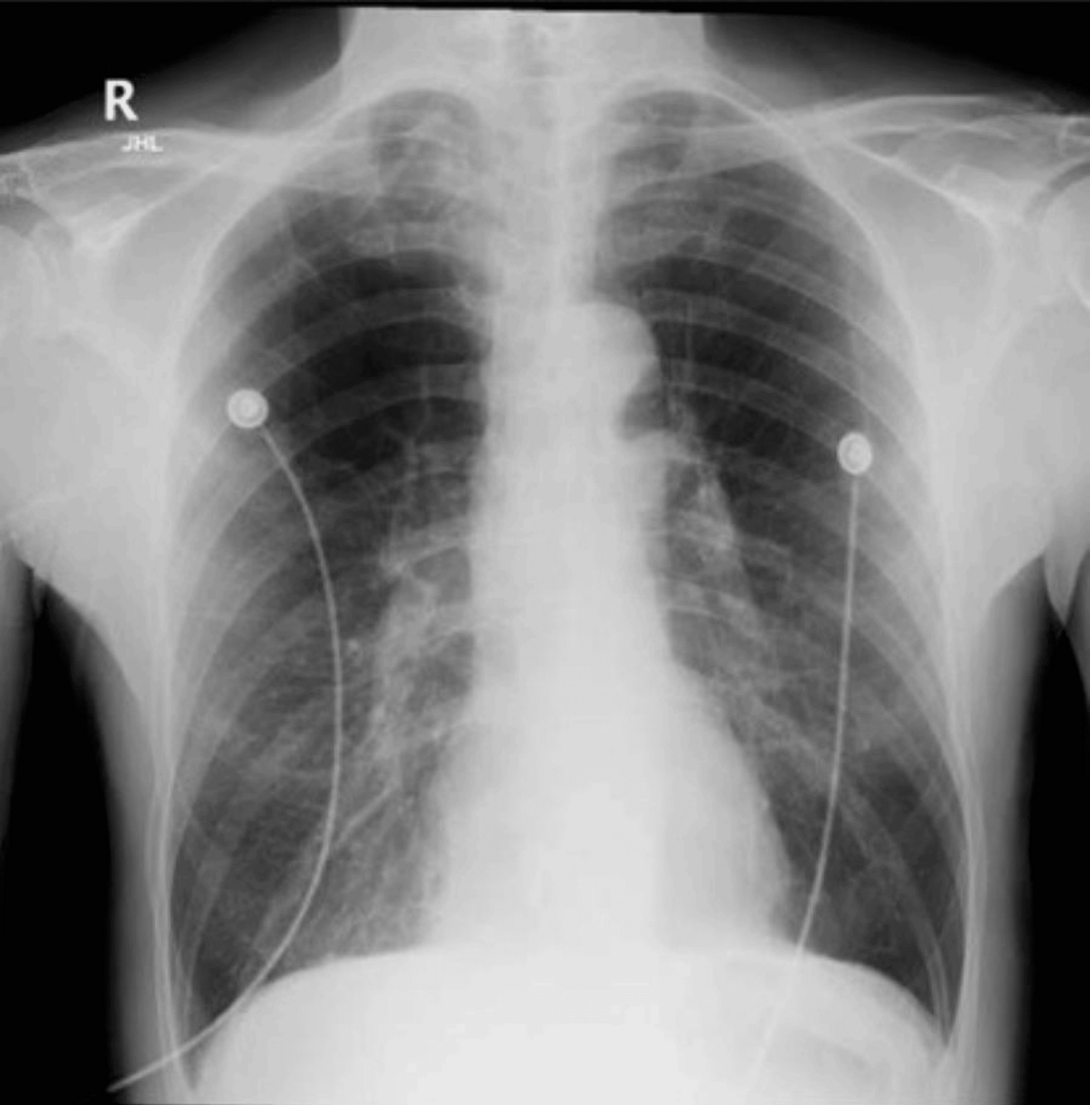
Chest radiograph in Case 1 demonstrating hyperinflation and diaphragmatic flattening An anteroposterior chest radiograph demonstrates hyperinflated lung fields with diaphragmatic flattening, supporting obstructive lung physiology in a patient with childhood-onset asthma, prior tobacco exposure, and emphysematous disease.

A 73-year-old man with childhood-onset asthma, COPD diagnosed several years earlier, chronic hypoxemic respiratory failure requiring 2 L/min of supplemental oxygen at home, hypertension, anxiety, and gastroesophageal reflux disease (GERD) was evaluated for progressive exertional dyspnea and intermittent wheezing. He was a former smoker with a 52-pack-year history and had quit smoking before the index pulmonary evaluation. Chest radiography demonstrated hyperinflation with diaphragmatic flattening (Figure 1), and high-resolution computed tomography of the chest performed during the same diagnostic period showed extensive bilateral emphysematous changes. Verified baseline and follow-up spirometry demonstrated persistent severe obstructive physiology, with minimal interval change between the included studies (Table 1).

**Table 1:** Baseline and follow-up spirometry in Cases 1-3 Forced vital capacity (FVC) and forced expiratory volume at one second (FEV1) are reported in liters (L), with percent predicted/reference in parentheses. FEV1/FVC is reported as the actual ratio in percent, followed by percent predicted/reference in parentheses. Peak expiratory flow (PEF) and forced expiratory flow between 25% and 75% of vital capacity (FEF 25-75) are reported in L/s. Forced expiratory time (FET) is reported in seconds when available. Only verified spirometry studies attributed to the respective patients are included.

Case and time point	FVC, L (% ref)	FEV1, L (% ref)	FEV1/FVC, actual % (% ref)	PEF, L/s (% ref)	FEF25-75%, L/s (% ref)	FET, sec	Interpretation/notes
Case 1: Baseline	1.96 (60.8%)	0.80 (32.3%)	41% (53.0%)	2.76 (37.9%)	0.28 (14.2%)	8.28	Severe obstructive defect.
Case 1: Follow-up 1	1.83 (57%)	0.82 (33%)	45% (58%)	2.07 (29%)	0.36 (18%)	--	Persistent severe obstruction.
Case 2: Baseline	2.02 (57.3%)	0.93 (33.5%)	46% (58.0%)	3.16 (45.2%)	0.37 (15.8%)	7.27	Severe obstructive defect. Actual FEV1/FVC is 46%; 58.0% is the percent predicted/reference.
Case 2: Month 1 follow-up	2.36 (66.9%)	1.32 (48.0%)	56% (71.2%)	4.42 (63.2%)	0.54 (22.8%)	7.78	Interval improvement in obstructive indices after initiation of inhaled therapy.
Case 3: Baseline	1.84 (68%)	0.97 (45%)	53% (66%)	2.54 (45%)	0.45 (21%)	--	Severe obstructive defect with marked expiratory flow limitation.
Case 3: Month 3 follow-up	2.24 (80%)	1.29 (58%)	58% (72%)	2.92 (50%)	0.62 (29%)	--	Interval improvement in obstructive indices after treatment escalation.

Laboratory evaluation demonstrated eosinophilia, with an absolute eosinophil count of 600 cells/µL, and markedly elevated total immunoglobulin E of 2,110 IU/mL. The combination of childhood-onset asthma, persistent obstructive physiology, heavy prior tobacco exposure, emphysema on imaging, eosinophilia, and elevated immunoglobulin E supported an asthma-COPD overlap phenotype with prominent type 2 inflammatory features. Because symptoms persisted despite optimized inhaled therapy, benralizumab was initiated for eosinophilic asthma.

Case 2

A 60-year-old woman with COPD diagnosed several years earlier, obesity, and hypertension presented with frequent exacerbations requiring multiple prednisone courses and limited functional capacity. She had an estimated 15-pack-year smoking history, smoking approximately one-half pack per day for 30 years, and had quit smoking before the index pulmonary evaluation. Baseline spirometry at the index evaluation showed severe obstruction, with a forced expiratory volume at one second (FEV1) of 0.93 L (33.5% predicted) and an actual FEV1/forced vital capacity (FVC) ratio of 46% (Table 1). She was started on fluticasone/salmeterol and tiotropium.

**Figure 2:**
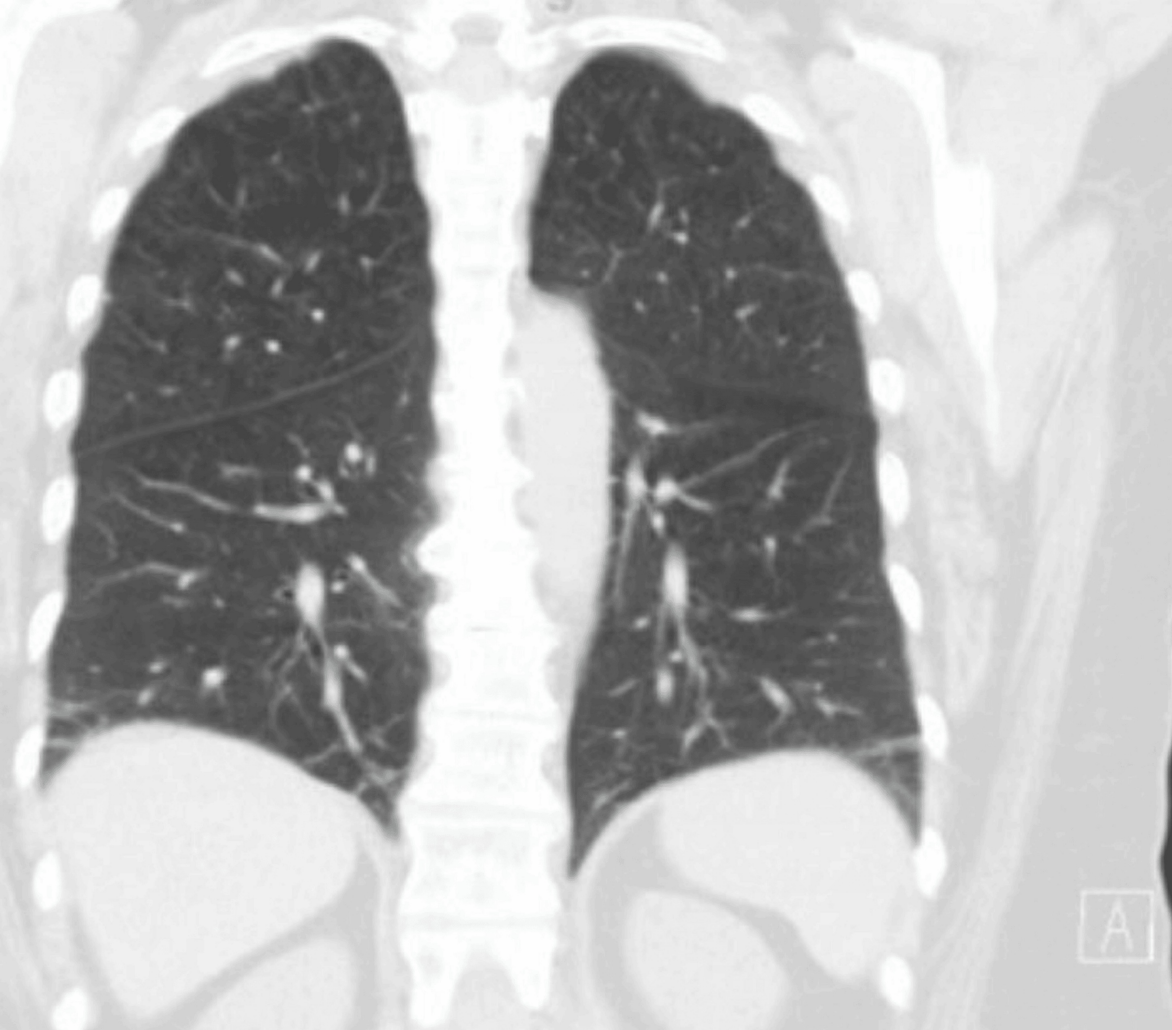
Coronal CT chest image in Case 2 demonstrating mild emphysematous change. A representative coronal CT chest image demonstrates mild emphysematous change, supporting COPD-associated radiographic features in the setting of obstructive spirometry and asthma-like inflammatory findings. COPD: chronic obstructive pulmonary disease

Computed tomography of the chest obtained shortly after the index evaluation demonstrated mild emphysema on a representative coronal image (Figure 2); the CT report also described a 5 mm right upper-lobe pulmonary nodule. At the month 1 follow-up, she reported substantial symptomatic improvement, and repeat spirometry demonstrated interval improvement, with FEV1 increasing to 1.32 L (48.0% predicted) and actual FEV1/FVC increasing to 56% (Table 1). Total immunoglobulin E was elevated at 401 IU/mL, and the absolute eosinophil count was 200 cells/µL. Allergy testing demonstrated sensitization to pecan, hickory, walnut, and cockroach. She was referred to allergy-immunology for further evaluation of asthma-like features and aeroallergen sensitization. The finalized allergy-immunology treatment plan was not available in the retrospective record reviewed for this case series.

Figure 6

Case 3

A 57-year-old woman with COPD diagnosed before the index pulmonary evaluation, allergic rhinitis, hypertension, hyperlipidemia, and GERD reported frequent rescue bronchodilator use, requiring a short-acting bronchodilator more than three times daily. She had an estimated 17.5-pack-year smoking history, smoking approximately one-half pack per day for 35 years, and had quit smoking before the index evaluation. Prior clinical documentation described a CT chest obtained before the index pulmonary evaluation as demonstrating mild upper-lobe-predominant centrilobular emphysema. Total immunoglobulin E was markedly elevated at 4,130 IU/mL, and allergy testing demonstrated sensitization to molds and dust mites.

**Figure 3:**
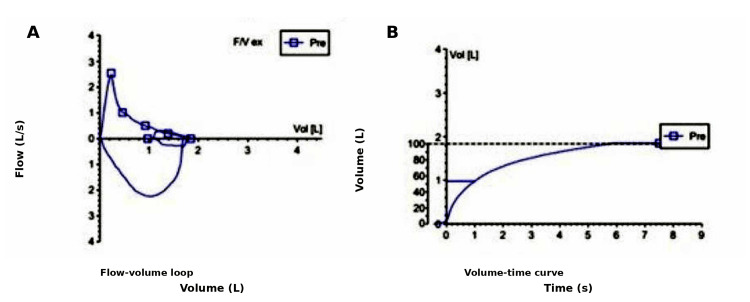
Initial flow-volume loop in Case 3 demonstrating expiratory flow limitation Panel A shows the baseline flow-volume loop, demonstrating a concave expiratory limb consistent with obstructive physiology. Panel B shows the corresponding volume-time curve from the same baseline spirometry study. Numeric spirometric values are summarized in Table 1.

Baseline spirometry showed severe obstruction, with FEV1 of 0.97 L (45% predicted) and actual FEV1/FVC of 53% (Table 1). The corresponding initial flow-volume loop demonstrated a concave expiratory limb and marked expiratory flow limitation, consistent with obstructive physiology (Figure 3). Given persistent symptoms despite maintenance inhaled therapy and prominent type 2 inflammatory features, dupilumab was initiated in combination with inhaled corticosteroid-containing therapy. The patient reported marked symptomatic improvement by the second dose. At the month 3 follow-up, repeat spirometry demonstrated interval improvement, with FEV1 increasing to 1.29 L (58% predicted) and actual FEV1/FVC increasing to 58% (Table 1). At a later pulmonary follow-up, she reported sustained improvement with reduced short-acting bronchodilator use while continuing dupilumab 300 mg every two weeks and maintenance inhaled therapy.

Figure 7

Case 4

**Figure 4:**
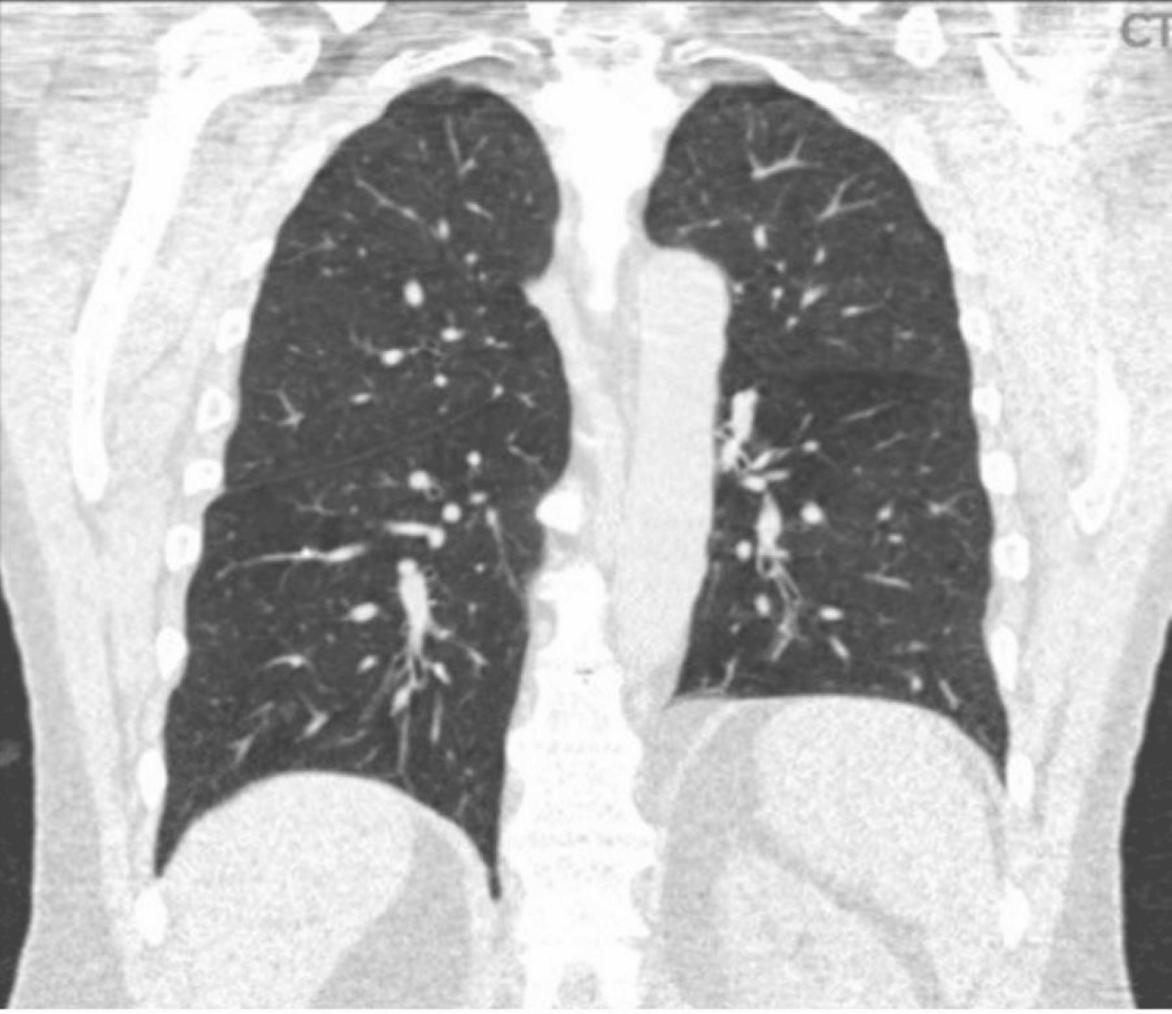
Coronal low-dose CT chest image in Case 4 demonstrating emphysematous changes A coronal low-dose CT chest image obtained before the index pulmonary evaluation demonstrates emphysematous changes, supporting COPD-associated radiographic features in a patient who also demonstrated bronchodilator responsiveness. COPD: chronic obstructive pulmonary disease

A 58-year-old man with COPD diagnosed several years earlier, coronary artery disease status post stenting, hypertension, obstructive sleep apnea treated with continuous positive airway pressure, and osteoarthritis was treated with inhaled corticosteroid/long-acting beta-agonist therapy and a long-acting muscarinic antagonist. After community-acquired pneumonia several months before the index pulmonary evaluation, he required home oxygen for three to four months before successful weaning. Low-dose computed tomography performed before the index evaluation demonstrated emphysematous changes (Figure 4).

**Figure 5:**
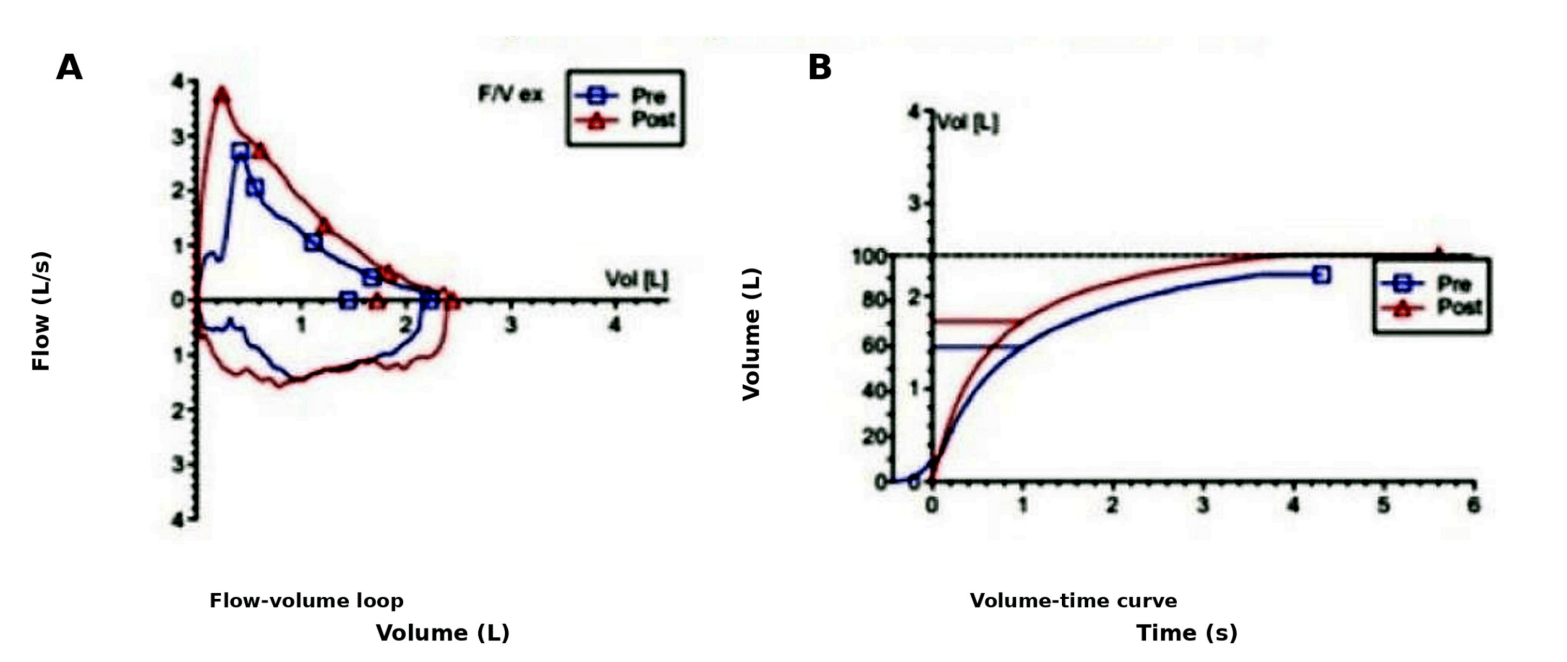
Pre- and post-bronchodilator flow-volume loops in Case 4 demonstrating bronchodilator responsiveness. Panel A shows pre- and post-bronchodilator flow-volume loops, demonstrating improved expiratory flow after bronchodilator administration. Panel B shows the corresponding pre- and post-bronchodilator volume-time curves. Quantitative spirometric values are summarized in Table 2.

Spirometry at the index pulmonary evaluation demonstrated obstructive physiology before bronchodilator administration, with FEV1 of 1.45 L (42% predicted) and FEV1/FVC of 65%. Post-bronchodilator testing showed improvement in FEV1 to 1.72 L (50% predicted), corresponding to an absolute increase of 270 mL and a relative increase of 19% (Table 2). The pre- and post-bronchodilator flow-volume loops demonstrated improved expiratory flow after bronchodilator administration (Figure 5). These findings support bronchodilator responsiveness and an asthma-like component. However, because the post-bronchodilator FEV1/FVC ratio increased to 71%, this specific test should not be presented as confirming persistent post-bronchodilator airflow obstruction. Chronic rhinitis prompted allergy evaluation, which demonstrated sensitization to multiple aeroallergens. Management remained focused on optimized inhaled therapy and clinical follow-up.

**Table 2:** Pre- and post-bronchodilator spirometry in Case 4 Forced vital capacity (FVC) and forced expiratory volume at one second (FEV1) are reported in liters (L). FEV1/FVC is reported as a percentage (%). Peak expiratory flow (PEF) and forced expiratory flow between 25% and 75% of vital capacity (FEF 25-75 are reported in L/s. These findings support bronchodilator responsiveness. LLN = lower limit of normal; pre-BD = pre-bronchodilator; post-BD = post-bronchodilator

Parameter	Reference	LLN	Pre-BD value	Pre % ref	Post-BD value	Post % ref	% change	Interpretation/notes
FVC (L)	4.40	3.37	2.23	51%	2.44	55%	+10%	Mild increase after bronchodilator.
FEV1 (L)	3.43	2.61	1.45	42%	1.72	50%	+19%	Absolute increase of 0.27 L; supports significant bronchodilator responsiveness.
FEV1/FVC (%)	78	66	65	83%	71	90%	+9%	Ratio improved after bronchodilator. Post-bronchodilator ratio is 71%.
PEF (L/s)	8.92	6.73	2.72	30%	3.77	42%	+39%	Improved peak expiratory flow.
FEF25-75% (L/s)	2.96	1.40	0.93	31%	1.22	41%	+32%	Improved mid-expiratory flow.

## Discussion

ACO is best understood as a heterogeneous clinical construct describing patients with chronic airflow limitation who demonstrate overlapping clinical, physiologic, radiographic, and inflammatory features of asthma and COPD, rather than as a single, discrete disease entity [7-9]. This interpretation is consistent with the 2024 GINA framing of this as a descriptive clinical term rather than a distinct disease, emphasizing that such patients may have worse outcomes than those with asthma or COPD alone and may require individualized assessment [2]. Systematic reviews suggest that patients with overlap features may experience greater symptom burden and increased exacerbation risk compared with patients with asthma or COPD alone, although reported estimates vary according to the diagnostic criteria used [4,6]. In this case series, an overlap phenotype was considered when COPD-associated features, including tobacco exposure where documented and radiographic emphysema coexisted with asthma-associated traits such as childhood asthma, bronchodilator responsiveness, allergic rhinitis or aeroallergen sensitization, eosinophilia, and elevated total IgE.

Therapeutic decisions in ACO are often extrapolated from asthma and COPD guidance because overlap populations remain underrepresented in prospective trials. In patients with asthma features, inhaled corticosteroids (ICS) remain central to treatment because of their established role in asthma control and exacerbation prevention [2]. In COPD, higher blood eosinophil counts have been associated with a greater likelihood of benefit from ICS-containing regimens, including reduced exacerbations in selected subgroups [10,11]. For COPD symptom control, long-acting bronchodilator therapy with a long-acting beta-agonist (LABA), long-acting muscarinic antagonist (LAMA), or combination therapy remains foundational, with escalation guided by symptom burden, exacerbation history, and inflammatory phenotype [3]. Importantly, LABA monotherapy should be avoided when asthma is present or strongly suspected because of safety concerns identified in the SMART trial [12]. When asthma remains uncontrolled despite ICS/LABA therapy, the addition of a LAMA may improve outcomes in selected adults [13]. Together, these principles support a phenotype-informed approach that accounts for the relative contribution of asthma-like inflammation, COPD-like fixed obstruction, emphysema, and exacerbation risk.

Evidence supporting biologic therapy in ACO remains limited and heterogeneous. Observational data suggest that some patients with ACO may exhibit biologic-response patterns comparable to those observed in severe asthma, supporting consideration of biologics in carefully selected patients with prominent type 2 inflammatory features and persistent symptoms or exacerbations despite optimized inhaled therapy [5]. However, other studies suggest that responses may be less favorable in ACO than in severe asthma alone, underscoring the need for careful patient selection and further prospective evaluation [14,15]. No individual biologic can currently be identified as superior for ACO because prospective head-to-head trials in standardized ACO populations are lacking. Biologic selection should therefore be phenotype- and endotype-informed, incorporating blood eosinophil count, total IgE, allergic sensitization, FeNO when available, comorbid type 2 inflammatory disease, exacerbation history, and the relative predominance of asthma-like versus COPD-like features. In the present case series, two patients with prominent type 2 features and persistent morbidity despite guideline-directed inhaled therapy were treated with benralizumab or dupilumab. The dupilumab-treated case demonstrated symptomatic improvement with objective spirometric improvement at three months. The benralizumab-treated case illustrates phenotype-guided biologic selection in eosinophilic asthma with coexisting emphysematous COPD features; however, after removal of an incorrectly included follow-up spirometry entry, the verified follow-up data show persistent severe obstruction with minimal interval change. These observations are hypothesis-generating only; causality cannot be inferred due to the uncontrolled design, small sample size, variable follow-up, and lack of standardized symptom scores, quality-of-life measures, or pre- and post-exacerbation rate comparisons.

Recent randomized data have expanded the evidence base for targeting type 2 inflammation in COPD, although these data remain indirect for ACO. In the BOREAS trial, dupilumab reduced moderate-to-severe exacerbations and improved lung function and quality-of-life measures in adults with COPD, chronic bronchitis, and elevated blood eosinophils despite standard therapy [16]. These findings were subsequently confirmed in the NOTUS trial [17]. The U.S. prescribing information identifies dupilumab as an add-on maintenance treatment for adults with inadequately controlled COPD and an eosinophilic phenotype, specifies subcutaneous administration every two weeks, and notes that dupilumab is not indicated for the relief of acute bronchospasm [18,19]. A recent systematic review and meta-analysis of type 2-targeted biologics in COPD summarized evidence from phase 2 and phase 3 randomized trials, including the broader trial context of dupilumab, mepolizumab, and benralizumab. In that analysis, dupilumab and mepolizumab consistently reduced moderate-to-severe exacerbations in eosinophilic or type 2 inflammatory COPD, whereas benralizumab showed no clear reduction; however, the authors cautioned that these findings derive from separate placebo-controlled trials and should not be interpreted as establishing a comparative efficacy hierarchy among biologics [20]. These data provide context for BOREAS and NOTUS for dupilumab, METREX/METREO and MATINEE for mepolizumab, and GALATHEA/TERRANOVA for benralizumab, but they do not establish comparative biologic efficacy in ACO [20]. Because these were COPD trials rather than ACO trials, direct generalizability to overlap phenotypes remains limited, and dedicated prospective studies in ACO populations are needed.

This case series has several limitations. First, ACO lacks a universally accepted diagnostic definition, and the included patients were identified using pragmatic clinical criteria rather than a standardized research definition. Second, the cases were drawn from clinical practice and are therefore subject to selection bias, variable follow-up, and incomplete data capture. Third, the small number of patients and heterogeneity of clinical presentations preclude conclusions regarding treatment efficacy. Fourth, FeNO values were not uniformly available, limiting objective assessment of type 2 airway inflammation across cases. Fifth, standardized patient-reported outcome measures, including the St. George’s Respiratory Questionnaire, COPD Assessment Test, and Asthma Control Test, were not uniformly available. Despite these limitations, the cases illustrate the practical value of integrating exposure history, spirometry, imaging, allergic evaluation, and accessible type 2 inflammatory biomarkers when evaluating patients with overlapping obstructive airway disease.

## Conclusions

Asthma-COPD overlap represents a heterogeneous clinical construct rather than a single uniform disease entity. This four-patient case series illustrates the diagnostic complexity that arises when persistent obstructive physiology and radiographic emphysema coexist with asthma-associated features, including bronchodilator responsiveness, allergic sensitization, eosinophilia, and elevated total immunoglobulin E. These cases support a phenotype-informed approach integrating clinical history, exposure assessment, spirometry, imaging, allergic evaluation, and accessible type 2 inflammatory biomarkers. In selected patients with persistent symptoms or exacerbations despite optimized inhaled therapy, biologic therapy may be considered as an individualized escalation strategy, although no specific biologic can currently be identified as superior for ACO. These observations remain descriptive and hypothesis-generating, and prospective studies using standardized diagnostic criteria, validated symptom and exacerbation measures, FeNO when available, and longitudinal pulmonary-function assessment are needed.
